# 

**Published:** 1921-06

**Authors:** 


A JOURNAL OF THE MEDICAL SCIENCES FOR THE
WEST OF ENGLAND AND SOUTH WALES.

*PUBLISHED UNDER THE AUSPICES OF*

*THE BRISTOL MEDICO-CHIRURGICAL SOCIETY.*

EDITOR:
P. WATSON-WILLIAMS, M.D.

WITH WHOM ARE ASSOCIATED

JAMES SWAIN, C.B., C.B.E., M.S., M.D., J. LACY FIRTH, M.S., M.D.
CAREY COOMBS, M.D.

J. A. NIXON, C.M.G., M.D., Assistant Editor.

EDITORIAL SECRETARY:
A. L. FLEMMING, M.B., Ch.B.

---

"Scire est nescire, nisi id me

Scire alius sciret."

---

Library of Congress

FEB 28 1922

SMITHSONIAN DEPOSIT

*VOL. XXXVIII.*

BRISTOL: J. W. ARROWSMITH LTD.
London: Simpkin, Marshall, Hamilton, Kent & Co. Limited.
1921.

### CONTENTS OF VOLUME XXXVIII.

| | Page |
|---|---|
| Some Problems in Surgical Treatment of Abdominal Conditions. By Forbes Fraser, C.B.E., F.R.C.S. .. .. .. .. | 1 |
| The Present Position of Psychotherapy. By R. G. Gordon, M.D., B.Sc., M.R.C.P. Ed. .. .. .. .. .. .. | 11 |
| The Teething Myth. By R. C. Clarke, O.B.E., M.B., B.Ch.Brist., M.R.C.P. .. .. .. .. .. .. .. .. | 28 |
| Remarks on Sinus Phlebitis and Thrombosis complicating Suppurative Middle Ear Disease. By J. P. I. Harty, B.A., M.B., B.Ch. (R.U.I.), F.R.C.S. .. .. .. .. .. | 73 |
| Progress in the Treatment of Empyema. By J. A. Nixon, C.M.G., M.D. (Cantab.), F.R.C.P. .. .. .. .. .. .. | 82 |
| A Case of Multiple Serositis (Pick's Disease) of Unusual Distribution. By H. J. Orr-Ewing, M.C., M.D., B.S. (Lond.), M.R.C.P. .. | 90 |
| Some Phases of Quackery in Relation to Diseases of the Eye. By Cyril H. Walker, M.B., B.A. Cantab. .. .. .. .. | 129 |
| The Repair of Bone Injuries. By Ernest W. Hey Groves, M.S., F.R.C.S. .. .. .. .. .. .. .. .. | 142 |
| The Rôle of Dilute Acids in Infection. By I. Walker Hall and A. D. Fraser .. .. .. .. .. .. .. .. | 158 |
| Progress of the Medical Sciences .. .. .. .. .. | 37 |
| Reviews of Books .. .. .. .. .. | 49, 94, 163 |
| Editorial Notes .. .. .. .. .. .. | 58, 118, 167 |
| The Library of the Bristol Medico-Chirurgical Society | 64, 125, 170 |
| Meetings of Societies .. .. .. .. .. .. .. | 66 |
| Obituary .. .. .. .. .. .. .. | 69, 127, 171 |
| Local Medical Notes .. .. .. .. .. | 72, 128, 172 |

J.W. Arrowsmith Ltd. Publishers Bristol

### The Library of the
### Bristol Medico-Chirurgical Society.

*The following donations have been received since the publication of the List in December.*

*April 30th*, 1921.

| | | |
|---|---|---|
| Dr. D. A. Alexander (1) | .. .. .. | 1 volume. |
| L. M. Griffiths (2) | .. .. .. .. | 1 ,, |
| Dr. J. R. Kay-Mouat (3) | .. .. .. | 9 volumes. |
| Medical Research Council (4) | .. .. .. | 1 volume. |
| Sir Humphry Rolleston, K.C.B. (5) | .. .. | 1 ,, |
| Dr. J. E. Shaw (6) | .. .. .. .. | 3 volumes. |
| Dr. Shingleton Smith (7) | .. .. .. | 2 ,, |

Unbound periodicals have been received from Mr. L. M. Griffiths, Mr. Hey Groves, Dr. James Swain and Mr. C. H. Walker.

### THE ONE HUNDRED AND THIRD LIST OF BOOKS.

The figures in brackets refer to the figures after the names of the donors, and show by whom the volumes were presented. The books to which no such figures are attached have either been bought from the Library Fund or received through the *Journal*.

**Bayliss, W. M.** .. *Principles of General Physiology* .. 3rd Ed. 1920

**Brockbank and A. Ramsbottom, E. M.** *Clinical Examination of Lung Diseases* .. .. .. .. .. .. .. 1921

**Cabot, R. C.** .. *Differential Diagnosis.* Vol. II. 2nd Ed. 1919

**Cannon, W. B.** .. *Bodily changes in Pain, Hunger, etc.* .. (3) 1915

*Catalogue* (*Index-*) *of the Library of the Surgeon-General's Office.* 3rd ser., Vols. I., II. .. .. .. .. .. 1918–20

**Clarke and E. E. Henderson, R. H.** *Investigation of the Central Nervous System* .. .. .. .. .. .. .. 1920

**Clubbe, C. P. B.** .. *Intussusception* .. .. .. .. .. .. 2nd Ed. 1921

**Crile, G. W.** .. .. *A Physical Interpretation of Shock, Exhaustion and Restoration* .. .. .. .. .. .. .. 1921

**Davis, H.** .. .. *Skin Diseases in General Practice.* [2nd Ed.] 1921

**Demoor, J.** .. .. *Cours de Physiologie générale* .. .. .. (3) 1905

**Emery, W. D.** .. *Clinical Bacteriology and Hæmatology.* 6th Ed. 1921

**Eve, Mrs. E. [Ed.]** *Manual for Health Visitors* .. .. .. .. .. 1921

**Ewart, E. D.** .. *A Guide to Anatomy* .. .. .. .. .. 1921

**Fuller, Sir B.** .. *The Science of Ourselves* .. .. .. .. .. 1921

**Gould, G. M.** .. *A Pocket Medical Dictionary* .. .. 8th Ed. 1920

Grenfell, W. T. .. *A Labrador Doctor* .. .. .. .. .. .. (1) [N.D.]

Hartley, Sir R. D. Powell and P. H.-S. *Diseases of the Lungs and Pleuræ* .. .. .. .. .. .. .. 6th Ed. 1921

Hay, J. .. .. .. .. *Graphic Methods in Heart Disease* [2nd Ed.] 1921

Henderson, R. H. Clarke and E. E. *Investigation of the Central Nervous System* .. .. .. .. .. .. .. 1920

Horder, Sir T. .. *Medical Notes* .. .. .. .. .. .. .. .. 1921

Humphris, F. H. .. *Electro-therapeutics* .. .. .. .. 2nd Ed. 1921

Jansen, M. .. .. *On Bone transformation* .. .. .. .. .. .. 1920

,, .. .. *Feebleness of Growth and Congenital Dwarfism* 1921

Johnston, J. .. .. *Musa medica* .. .. .. .. .. .. .. (2) 1897

Lister, Lord .. .. *Six Papers by* (Notes by Sir R. J. Godlee) .. 1921

McLeod, J. J. R. .. *Physiology and Biochemistry in Modern Medicine* .. .. .. .. .. (6) 3rd Ed. 1920

Manson, Sir P. .. *Tropical Diseases* (Ed. by P. H. Manson-Bahr) .. .. .. .. .. .. .. .. 7th Ed. 1921

Martindale and W. W. Westcott, W. H. *The Extra Pharmacopœia.* Vol. II. 17th Ed. 1921

Maublanc and Ratié. *The Medical Examination of Airmen* (Trans. by N. Ball) .. .. .. .. .. .. .. .. .. 1920

*Mediterranean War Area, Some Medical Diseases in the* .. .. (3) 1917

*Military Hygiene, Manual of Elementary* .. .. .. .. .. .. (3) 1912

Mosso, A. .. .. *La Peur* .. .. .. .. .. .. (3) 3[me] Éd. 1905

,, .. .. *La Fatigue* .. .. .. .. .. (3) 5[me] Éd. 1905

Paget, S. .. .. *Sir Victor Horsley* .. .. .. .. .. .. (7) 1919

Parker, G. .. .. *Early History of Surgery in Great Britain* .. 1920

Powell and P. H.-S. Hartley, Sir R. D. *Diseases of the Lungs and Pleuræ* .. .. .. .. .. .. .. 6th Ed. 1921

Price-Jones, C. .. *Blood-Pictures* .. .. .. .. .. .. 2nd Ed. 1920

Ramsbottom, E. M. Brockbank and A. *The Clinical Examination of Lung Diseases* .. .. .. .. .. .. .. .. 1921

Ratié, Maublanc and *The Medical Examination of Airmen* (Trans. by N. Ball) .. .. .. .. .. .. 1920

Reuss, A. Ritter von *The Diseases of the Newborn* (Ed. by J. D. Rolleston) .. .. .. .. .. .. .. .. .. 1921

Riviere, C. .. .. *Early Diagnosis of Tubercle* .. .. 3rd Ed. 1921

Rolleston, Sir H. *Lumleian Lectures on Cerebro-Spinal Fever* (5) 1919

Taylor [A. S.] .. *Principles and Practice of Medical Jurisprudence* (Ed. by F. J. Smith) (6) 7th Ed. 1920

Thomson, J. A. .. *Outlines of Zoology* .. .. .. .. 7th Ed. 1921

Tunstall, F. J. Warwick and A. C. *First Aid* .. .. 11th Ed. 1920

Turner, A. L. .. *Sir William Turner* .. .. .. .. (7) 1919

Warwick and A. C. Tunstall, F. J. *First Aid* .. .. 11th Ed. 1920

Westcott, W. H. Martindale and W. W. *The Extra Pharmacopœia.* Vol. II. 17th Ed. 1921

Wilson, R. M. .. *The Care of Human Machinery* .. .. .. 1921

*Wound Infections, Studies in* (Med. Res. Council) .. .. .. (4) 1920

TRANSACTIONS, REPORTS, JOURNALS, &c.

American Laryngological Association, Transactions of the .. .. 1918
American Laryngological, Rhinological, and Otological Society, Transactions of the .. .. .. .. .. .. .. .. .. .. .. 1920
American Ophthalmological Society, Transactions of the Vol. XVIII. 1920
American Pediatric Society, Transactions of the.. Vol. XXXII. 1920
Edinburgh Obstetrical Transactions .. .. .. .. .. Vol. XL. 1920
Hospitals—St. Bartholomew's Hospital Reports .. Vol. LIII. 1920
London Dermatological Society, Transactions of .. .. .. .. 1920
Medical Society's Transactions .. .. .. .. .. .. Vol. XLIII. 1920
Smithsonian Report for 1918 .. .. .. .. .. .. .. .. .. .. 1920
Studies from the Department of Pathology of the College of Physicians and Surgeons, N.Y. .. .. .. .. .. Vol. XVII. 1920

### MEETINGS OF SOCIETIES.

UNIVERSITY OF BRISTOL.—*M.D.:* A. R. Heber, A. D. Symons.

*M.B., Ch.B.:* W. H. Royal, Marjorie Wadsworth (1st Class Honours).

*Final Examination, Part I.:* P. Beames, M. Critchley, J. R. Duerden, W. G. Nott, J. A. Pritchard, W. K. A. Richards, V. S. Tryon. (*Including Forensic Medicine and Toxicology*) *:* W. A. Jackman, P. Phillips.

*Second Examination* (*Elementary Anatomy*) *:* C. F. Killick, A. C. Lysaght, E. May, E. S. Rogers.

*Second Examination, Part II.:* M. C. Barclay, S. H. Blacker, A. P. Bodman, N. J. Bown, E. G. Bradbeer, G. B. Bush, E. R. Clutterbuck, W. L. Cossham, M. F. Dalton, H. M. Dixon, R. H. Dummett. L. Dunn, F. R. Edbrooke, E. B. Eedle, N. J. England, H. M. Golding, W. A. Gornall, J. L. Griffin, R. C. Hatcher, A. G. Heron, J. A. James, F. G. Jenkins, D. E. Joscelyne, E. F. King, A. H. Marshall, J. R. Nicholson-Lailey, A. S. Prowse, R. A. Sammons, A. E. Sherwell, H. Taylor, R. E. Whitby, C. F. Wilson, G. S. Mundy.

*D.P.H., Part II.:* F. V. Cant, A. D. Fraser, G. C. Williams.

*Diploma in Dental Surgery.—First Examination:* S. H. Bedford, J. G. Bishop, W. B. Bowrey, M. P. Browning, A. W. Cawsey, P. O. Dyer, C. H. Hill, H. F. D. Lane, R. Lonnon, E. W. Mogg, R. C. Price, J. F. Rigg, J. S. Roberts, B. F. Robinson, C. F. M. Taylor.

*Second Examination:* R. March. *Part I.:* J. Evans, H. C. Hopes, K. A. Lewis, E. H. Longley, C. G. Lewis, C. G. Payne, H. W. Pidgeon, G. G. Royal, C. G. Saxon, C. E. Smith, G. A. Thomas, C. R. Woods.

*Third Examination* (*Dental Anatomy*) *:* R. H. Davies, H. C. Hopes, K. A. Hunt, E. H. Longley, C. G. Payne, H. W. Pidgeon, M. Ritblatt, C. R. Woods.

*Final Examination:* N. H. Bodenham.

CONJOINT EXAMINING BOARD OF ENGLAND.—*Anatomy and Physiology:* F. H. Woolley, E. Butler, C. F. Powell.

*Materia Medica:* M. A. Summers.

